# Lateral extra‐articular procedures combined with ACL reconstructions lead to a higher return to pre‐injury level of sport: A systematic review and meta‐analysis

**DOI:** 10.1002/jeo2.70196

**Published:** 2025-03-11

**Authors:** Guus Felix Kerkvliet, Gijs Bram Peter Cornelis van der Ree, Inger Nicoline Sierevelt, Gino M. M. J. Kerkhoffs, Bart Muller

**Affiliations:** ^1^ Faculty of Medicine, Amsterdam University Medical Centre, University of Amsterdam Amsterdam the Netherlands; ^2^ Department of Orthopedic Surgery Xpert Clinics Amsterdam the Netherlands; ^3^ Department of Orthopedic Surgery Spaarnegasthuis Academy Hoofddorp the Netherlands; ^4^ Department of Orthopedic Surgery and Sports Medicine Amsterdam University Medical Centre, Academic Centre for Evidence-Based Sports Medicine (ACES) Amsterdam the Netherlands

**Keywords:** ACL reconstruction, lateral extra‐articular procedure, lateral extra‐articular tenodesis, postoperative activity level, return to pre‐injury level sport, return to sport

## Abstract

**Purpose:**

To compare postoperative activity levels between patients who received an anterior cruciate ligament reconstruction (ACLR) with‐ and without a lateral extra‐articular procedure (LEAP).

**Objectives:**

The primary objective is to examine whether patients treated with an ALCR and LEAP have a greater chance to return to sport (RTS) and return to their pre‐injury level of sport (RTPS). The re‐rupture rates between the two groups will also be analysed as this is of great influence on the RTS and RTPS.

**Methods:**

A thorough search according to PRISMA guidelines was conducted through the PubMed and Embase databases in May 2024. Randomised controlled trials (RCT) and retrospective cohort studies on patients who underwent primary ACLR with‐ or without a LEAP were included. Postoperative Tegner score, RTS, RTPS and re‐rupture rate were evaluated. All articles were revised according to Cochrane risk of bias tools (RoB 2.0 and ROBINS‐I).

**Results:**

Twenty‐four studies were included after examining 966 titles, abstracts and manuscripts. A total of 33,527 patients were included in this review with a weighted mean age of 24.9 years. Pooled data demonstrates that the ACLR + LEAP group shows significantly higher postoperative Tegner scores (MD, 0.43 [95% confidence interval, 0.21–0.65]; *p* < 0.01). 62% of patients who underwent ACLR + LEA returned to their pre‐injury level of sport compared to 40% in ACLR group (reported in nine studies).

**Conclusion:**

This meta‐analysis demonstrates that patients undergoing a LEAP procedure in addition to ACLR return to higher postoperative activity levels and are more likely to return to their pre‐injury level of sport. These results ‐in addition to further research‐ may help dictate when to add a LEAP, and whether LEAP in addition to ACLR should become the golden standard.

**Level of Evidence:**

Level III, retrospective cohort studies have been analysed, alongside RCT's, and thus this is the level of evidence.

**Abbreviations**
95% CI: 95% confidence interval
ACL: anterior cruciate ligament
ACLR: anterior cruciate ligament reconstruction
ALC: anterolateral complex
ALL: anterolateral ligament
ALSA: anterolateral structure augmentation
ITB: iliotibial band
ITT: intention to treat
LEAP: lateral extra‐articular procedure
LET: lateral extra‐articular tenodesis
LOE: level of evidence
MD: mean difference
non‐RCT: non‐randomised controlled trial/retrospective cohort study
PRISMA: Preferred Reporting Items for Systematic Reviews and Meta‐analyses
RCT: randomised controlled trial
RoB: risk of bias
RR: risk ratio
RTPS: return to pre‐injury‐level sport
RTS: return to sport
ST: semitendinosus

## INTRODUCTION

The prevalence of anterior cruciate ligament (ACL) injury is the highest in pivoting‐sports with high‐impact rotational landings as a part of the sports nature [43]. Rupturing the ACL results in instability of the knee joint which may leave the athletes out of the game for a prolonged period of time [31]. With regards to surgical treatment, ACL‐reconstruction (ACLR) has long been‐ and is‐ the golden standard. However, a growing body of literature suggests that controlling rotatory laxity may not be sufficiently achieved [16]. Since rotatory stability is an absolute necessity when pivoting, not achieving this has an extensive impact on whether patients can return to their (pre‐injury level of) sport [2]. For athletes, persistent rotatory instability causes lower‐ and delayed return to sport (RTS) rates [2].

Several biomechanical studies have suggested that the anterolateral complex (ALC) of the knee contributes to rotatory instability [37, 48]. Lateral extra‐articular procedure (LEAP) is a collective name for surgical procedures on the ALC, commonly performed in addition to ACLR. The exact technique may vary between these surgical interventions [15, 21, 29, 33, 38]. Mao et al. [40] reported a 56% risk reduction for anterolateral rotatory knee instability among patients who received an ACLR combined with lateral extra‐articular tenodesis (LET), a type of LEAP.

Numerous reviews focus on the different technical aspects of LEAPs in addition to ACLR [8, 13, 22, 32, 40]. However, little is known about its clinical implications with regards to RTS and return to pre‐injury sport (RTPS) and then ‐more specifically‐ pivoting sports. Since LEAPs seem to contribute to the rotatory stability of the knee [29], patients that partake in sports ‐and predominantly pivoting sports‐ hypothetically benefit most from these procedures [1]. Pivoting sports are defined as sports where fast start‐ and stop and rotational movements are part of the nature of the sport [62].

Therefore, the aim of this study is to review the role of LEAPs in addition to ACLR with regards to the postoperative activity level. The hypothesis is that LEAPs lead to higher postoperative activity levels and higher RTS‐ and RTPS‐rates as in vitro studies have suggested that the ALC plays a critical role in controlling rotational instability [37].

## METHODS

### Databases and search strategy

This systematic review was completed according to the PRISMA statement [47]. A thorough search was conducted through the PubMed and Embase databases in May 2024, with the following search string: ‘Anterior cruciate ligament injuries’, ‘ACL‐reconstruction’, ‘lateral extra‐articular tenodesis’, ‘anterolateral ligament reconstruction’, ‘anterolateral augmentation’, ‘activity level’ and ‘return to sport’. The complete search can be found in Supporting Information: S1.

### Selection process and eligibility criteria

Two independent reviewers (G.K. and G.R.) systematically reviewed studies that compared primary ACLR with‐ and without a concomitant LEAP. Titles and abstracts were screened to exclude irrelevant articles. The remaining articles were screened for full‐text eligibility and examined using the inclusion‐ and exclusion criteria. Both reviewers recorded their included‐ and excluded articles, together with exclusion reasons, in Rayyan (Cambridge, Massachusetts, USA) software [46]. Within this software we filtered the results based on language and study design. A third reviewer (B.M.) was consulted in case of disagreement between the two reviewers.

Eligible studies met the following criteria: (1) the study had to involve patients that underwent an isolated primary ACLR and a primary ACLR in combination with a LEAP; (2) ACLR technique and graft choice had to be identical in the intervention‐ and control group (ensuring the only difference was the addition of a LEAP); (3) the Tegner score [58] and/or RTS and/or return to pre‐injury level of sport (RTPS) was defined as outcome and (4) with a minimum follow‐up period of 1 year. Exclusion criteria were: (1) articles in non‐English; (2) articles on revision ACL‐surgery and (3) articles published prior to 2010. In order to provide an up‐to‐date review and analysis, and to avoid the inclusion of potentially outdated surgical techniques, literature prior to 2010 was excluded from this review.

### Data extraction

Extracted study characteristics included: author, country, publication year, study design, level of evidence (LOE), number of patients, mean age, mean follow‐up period and brief patient characteristics and study groups. Furthermore, the percentage of patients involved in pivoting sports was also collected where possible. Surgical details of each article included: ACLR‐technique, graft type and LEAP‐technique. Only the postoperative Tegner score was collected. Although comparison with pre‐injury activity levels would greatly help further interpreting these results, the pre‐injury activity levels were not presented in most included studies. Thus, only the postoperative Tegner score was collected, as advised by Cochrane when drop‐outs are present in most studies [24]. Furthermore, the RTS and RTPS were also extracted as *primary outcome* where possible. The respective author of each study was contacted if the relevant data was presented with a median instead of a mean, so that data could be pooled. If no response was received from the respective authors, medians were converted into means (and standard deviation) according to data management techniques by Hozo et al. [26] and Walter and Yao [63]. In case sample sizes exceeded 25, the median itself was the best estimator of the mean [26]. Standard deviations were extrapolated from the range by utilising a conversion factor which is dependent on the sample size [63]. Graft re‐rupture defined as the postoperative rupture of the operated ACL which required revision ACL‐surgery was collected as secondary outcome. Data was collected and organised in a Microsoft Excel (Microsoft, Redmond, Washington, USA) spreadsheet.

### Critical appraisal

As this systematic review includes both RCTs and non‐RCTs, two different tools were utilised to determine the risk of bias of the included studies according to checklists formulated by the Cochrane Institute. The RoB 2.0 tool [25] and the Robins‐I tool [56] were used to assess the included RCT's and non‐RCT's, respectively. The algorithms of both tools classify the domains on the included studies as low risk of bias (+); some concerns/moderate (−); or high/serious risk of bias (X). Combining the conclusions of all domains per article generates an overall risk of bias level for each respective article.

### Statistical analysis

To assess differences between the ACLR and ACLR with LEAP, meta‐analyses were carried out based upon mean differences (MD) with their respective 95% confidence intervals (CI) for the postoperative Tegner score. Risk ratios (RR) were calculated with their 95%CI for dichotomous outcomes, such as the RTS, RTPS and re‐rupture rate. Data were pooled by use of random effect model with inverse variance weighting. Subgroup analyses were performed to compare differences of the pooled effects between patients participating in pivoting and non‐pivoting sports using Q‐tests. A study population was considered to participate in pivoting sports in case ≥80% of the patients participated in pivoting sports. This threshold was chosen arbitrarily to create larger subgroups for analysis. Sensitivity analyses for RTS and RTPS were performed to examine the effect of any missing results as some studies reported data lost to follow‐up. Additional sensitivity analyses for all outcomes were utilised to investigate whether the included non‐RCTs presented similar results as the RCTs. Potential publication bias was assessed by use of a Funnel plot for the Tegner scale. Heterogeneity between studies is expressed by the *I* ^2^‐value and interpreted according to the Cochrane handbook [23]. An *I* ^2^‐value less than 50% represents low‐moderate heterogeneity; an *I* ^2^‐value between 50% and 75% represents substantial heterogeneity; and an *I* ^2^‐value above 75% represents considerable heterogeneity. Rstudio (Boston, Massachussets, USA) version 2022.07.1, package Meta version 5.5‐0 [6] was utilised for all meta‐analyses.

## RESULTS

### Study selection

**Figure 1:**
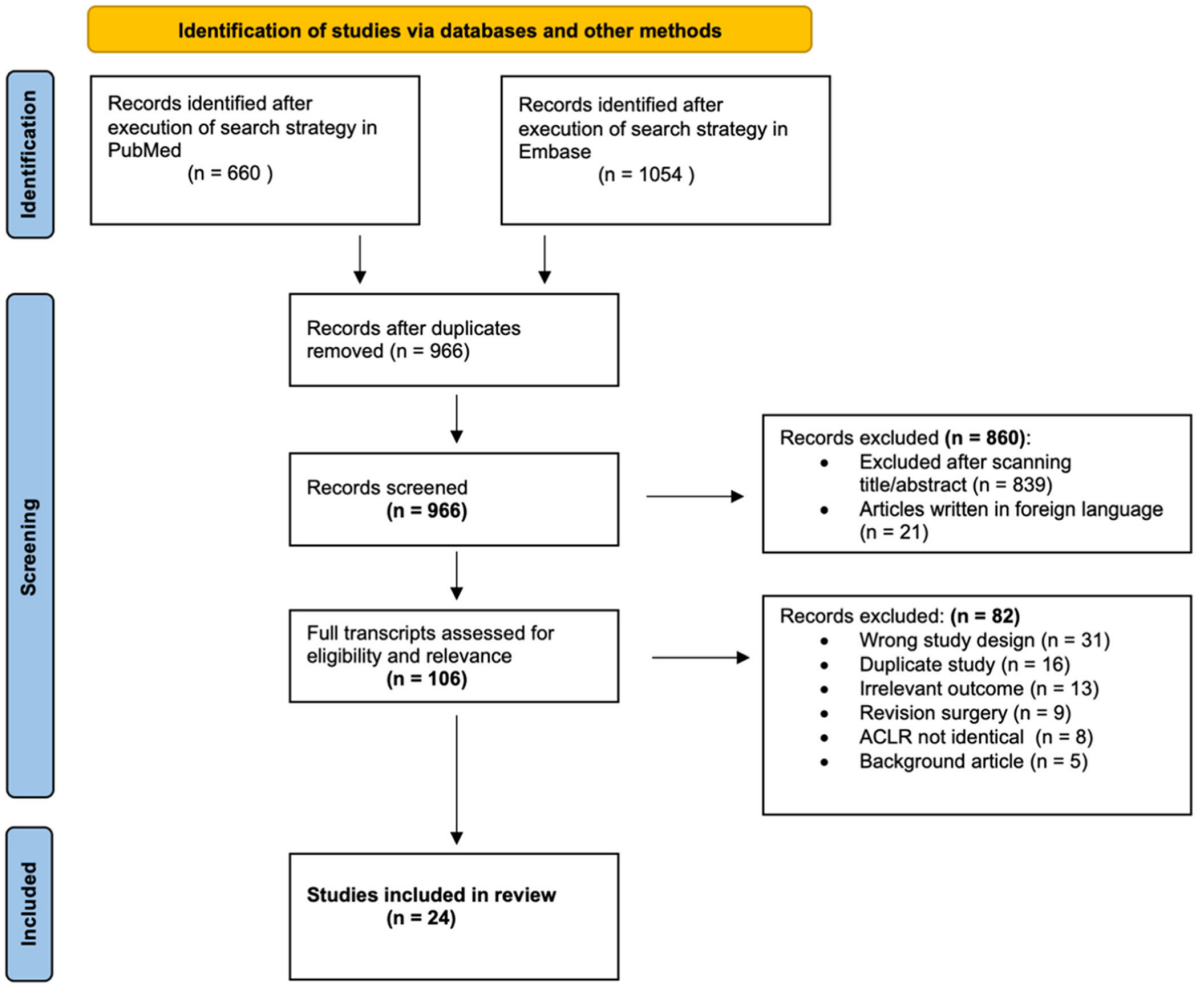
PRISMA flow diagram illustrating the systematic process of including articles.

The search generated 1714 articles of which 966 remained after the duplicate removal. After screening titles and abstracts for relevance, 106 articles were comprehensively reviewed with emphasis on the reported outcomes and their comparative nature. Eight studies adhered to the inclusion criteria but were excluded because the ACLR‐technique was not identical in the intervention‐ and control group. The reasons for excluding another 82 articles are presented in Figure 1. After a thorough selection process, 24 articles [9, 12, 18, 20, 27, 28, 30, 34, 36, 39, 41, 42, 49, 51, 53, 54, 59, 60, 64, 65] were included in this review and meta‐analysis.

### Methodological quality

**Figure 2:**
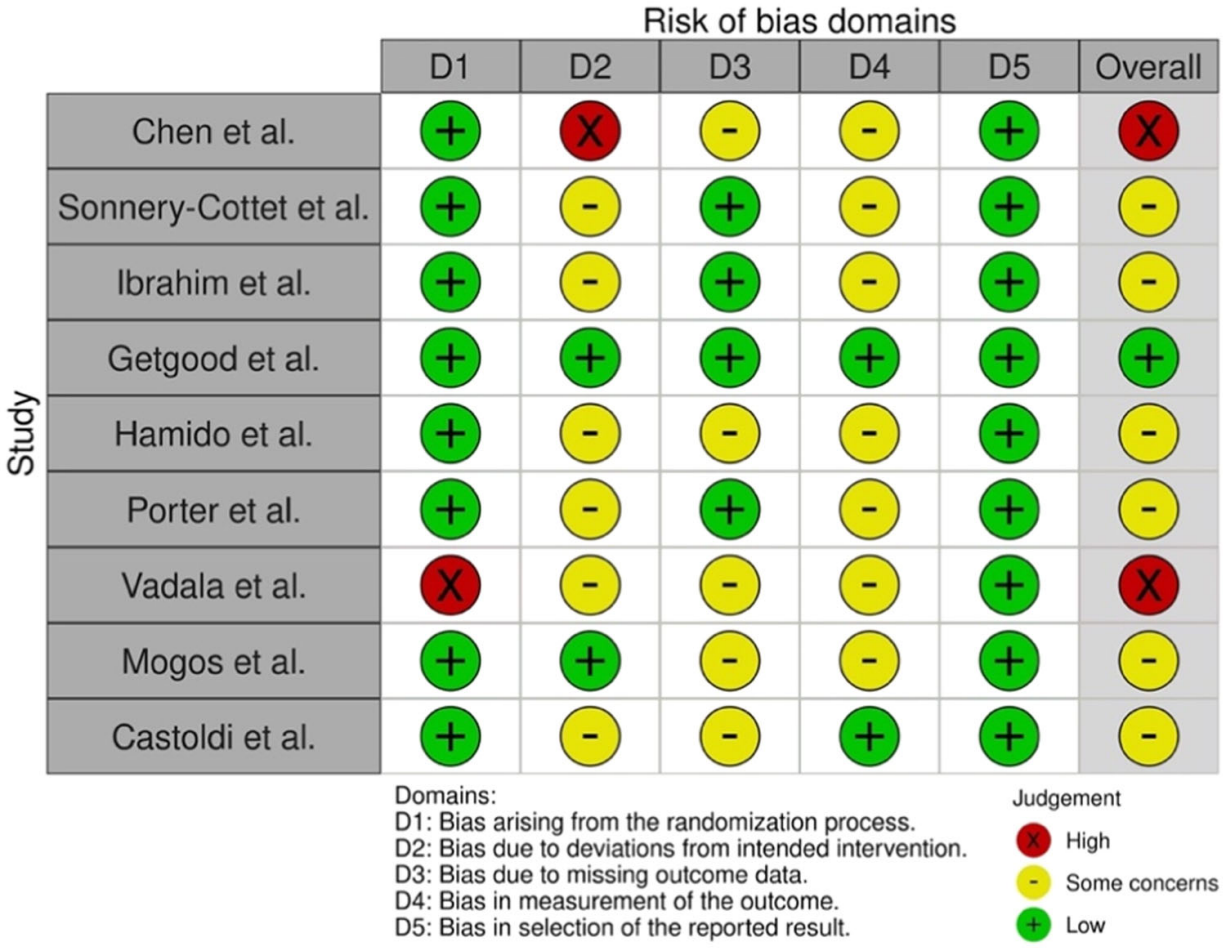
Critical appraisal of the included randomised controlled trials according to the RoB2 checklist. RoB, risk of bias assessment.

**Figure 3:**
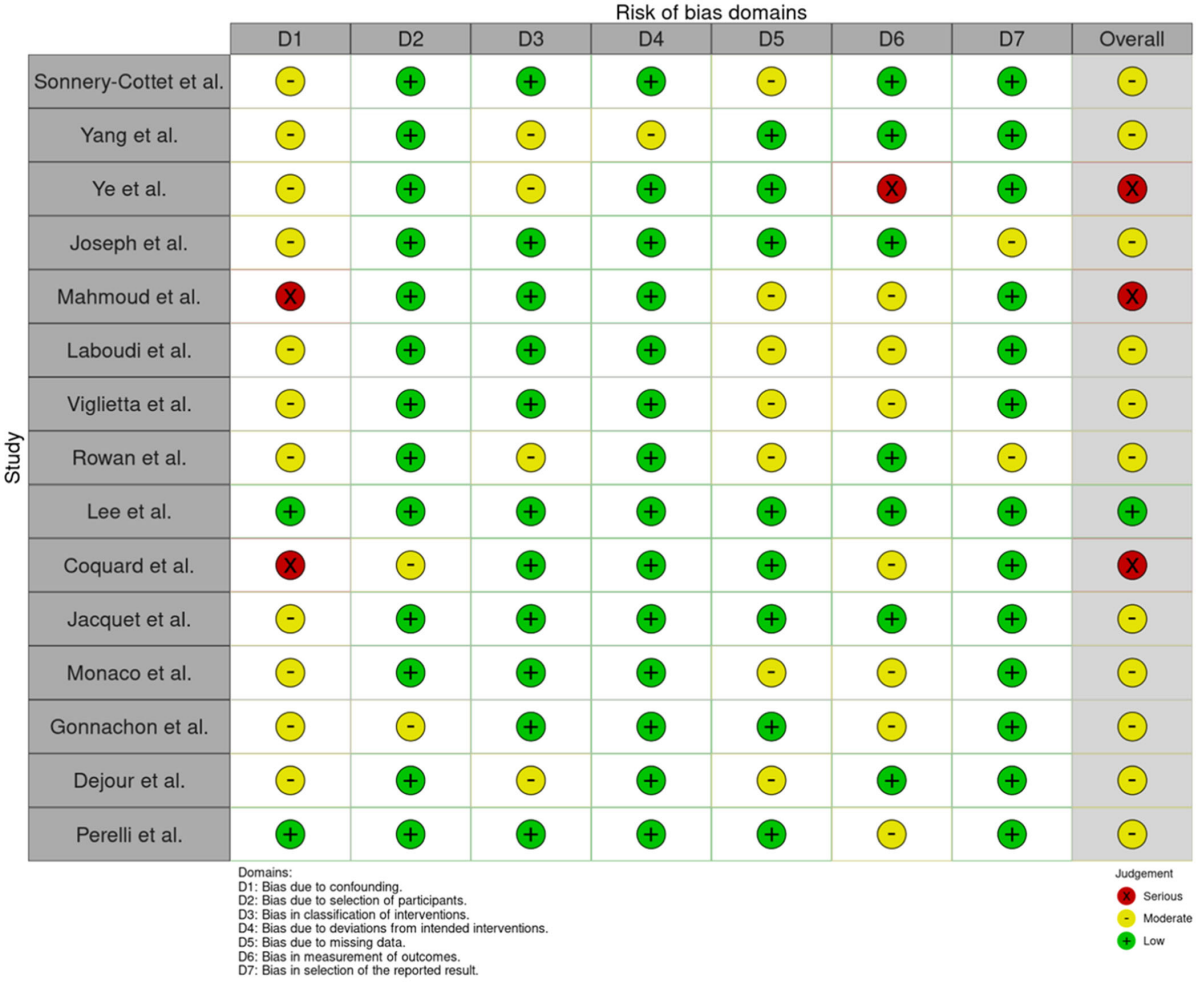
Critical appraisal of the included non‐randomised controlled trials according to the ROBINS‐1 checklist.

The RoB results according to the Cochrane Institute checklists are presented in Supporting Information: S1 (Figures 2 and 3). Most of the RCTs contained ‘*some concerns’* regarding the risk of bias. One RCT [18] was deemed to be of very high quality and two RCTs [10, 59] were deemed to be of a lower quality. Most of the non‐RCTs received an overall score of *moderate* risk of bias. A trend across some of these articles was selection bias which will be addressed later.

### Study characteristics

A total of 3527 patients were included in this review. Nine out of the 24 included articles were RCTs and the remainder were retrospective comparative studies and cohort studies. The weighted mean age of the included patients was 24.9 years. Eleven articles [9, 10, 20, 28, 34, 41, 50, 53, 54, 64, 65] studied patients partaking in pivoting sports, which were thus included in the subgroup analysis for pivoting sports. Table 1 shows a summary of study characteristics.

**Table 1:** Study characteristics.

Study (year)	Country	Study design	LOE	*N*	Mean age	Mean follow‐up	% pivoting *N*	Patients' characteristics	Groups
Sonnery‐Cottet et al. [54]	France	Cohort study	III	397	22 ± 4.0	38 ± 8.5	100%	Primary unilateral ACL‐rupture & participating in pivoting sports	ACLR‐4HT (*n* = 176) ALCR‐4HT + ALLR (*n* = 221)
Yang et al. [64]	South‐Korea	Retrospective comparative study	III	70	26 ± 10	48 ± 17	100%	Primary unilateral ACL‐rupture & participating in sports	ACLR (*n* = 35) ACLR + ALLR (*n* = 35)
Ye et al. [65]	China	Retrospective comparative study	III	92	29 ± 8.1	NR	88.0%	Primary unilateral ACL‐rupture & participating in sports	ACLR (n= 44) ACLR + ALSA (n= 48)
Chen et al. [10]	China	Randomised Controlled Study	II	120	30 ± 6.7	25 ± 1.4	90.6%	Primary unilateral ACL‐rupture & participating in sports	ACLR (*n* = 57) ACLR + ALSA (*n* = 63)
Joseph et al. [30]	France	Retrospective comparative study	III	87	29 ± 6.5	NR	NR	Primary unilateral ACL‐rupture & participating in sports	ACLR (*n* = 52) ACLR + Lemaire (*n* = 35)
Sonnery‐Cottet et al. [53]	France	Randomised Controlled Study	II	224	25 ± 4.4	12 ± 1.9	86.2%	Patients (18‐35) with primary unilateral ACL‐rupture & participating in sports	ACLR (*n* = 112) ACLR + ALLR (*n* = 112)
Ibrahim et al. [27]	Kuwait	Randomised Controlled Study	II	103	26 ± NR	27 ± NR	NR	Primary unilateral ACL‐rupture	ACLR (*n *= 50) ACLR + ALLR (*n* = 53)
Getgood and Moatshe [17]	Canada	Randomised Controlled Study	II	618	19 ± NR	24 ± NR	NR	Patients (14–25) with primary unilateral ACL‐rupture & participating in sports	ACLR (*n* = 312) ALCR + LEAT (*n* = 306)
Hamido et al. [20]	Kuwait	Randomised Controlled Study	II	102	25 (18‐40)*	60 (55‐65)*	100%	Primary unilateral ACL‐rupture & participating in pivoting sports	ACLR (*n* = 52) ACLR + ALLR (*n* = 50)
Mahmoud et al. [39]	Australia	Retrospective comparative study	III	144	25 ± 8.4	60 (NR)*	NR	Primary unilateral ACL‐rupture	ACLR (*n *= 72) ALCR + LEAT (*n* = 72)
Laboudi et al. [34]	France	Retrospective comparative study	III	203	16 ± 2.0	58 ± 11	100%	Primary unilateral ACL‐rupture & participating in pivoting sports.	ACLR (*n* = 101) ACLR + ALLR (*n* = 102)
Viglietta et al. [60]	Italy	Cohort study	III	165	27 ± NR	188 ± NR	NR	Patients <50 with primary unilateral ACL‐rupture	ACLR (*n* = 79) ALCR + LEAT (*n* = 86)
Rowan et al. [51]	UK	Retrospective comparative study	III	171	28* (NR)	NR	NR	Primary unilateral ACL‐rupture & participating in sports	ACLR (*n* = 125) ALCR + LEAT (*n* = 46)
Lee et al. [36]	South‐Korea	Retrospective comparative study	III	78	31 ± 5.9	30 ± 3.7	NR	Primary unilateral ACL‐rupture & grade ≥2 pivot shift	ACLR (*n* = 39) ACLR + ALLR (*n* = 39)
Coquard et al. [11]	France	Retrospective comparative study	III	222	38 ± NR	NR	NR	Primary unilateral ACL‐rupture & experienced subjective knee instability	ACLR (*n* = 111) ACLR + ALLR (*n* = 111)
Porter and Shadbolt [50]	Australia	Randomised Controlled Study	II	55	22 ± 3.9	NR	100%	Skeletally mature patients with primary unilateral ACL‐rupture & participating in pivoting sports	ACLR (*n* = 27) ACLR + MITBT (*n* = 28)
Vadala et al. [59]	Italy	Randomised Controlled Study	II	60	27 ± NR	44 ± NR	NR	Primary unilateral ACL‐rupture & +2 or +3 grade pivot shift.	ACLR (*n* = 32) ALCR + LEAT (*n* = 28)
Mogos et al. [41]	Romania	Randomised Controlled Study	II	57	31 ± 7.1	NR	100%	Primary unilateral ACL‐rupture & participating in pivoting sports.	ACLR (*n* = 25) ACLR + ALLR (*n* = 32)
Jacquet et al. [28]	France	Cohort Study	III	132	31 ± 8.2	44 ± NR	100%	Primary unilateral ACL‐rupture, no collateral ligament injury & +2 or +3 grade pivot shift.	ACLR (*n* = 55) ACLR + LEAT (*n* = 77)
Monaco et al. [42]	Italy	Retrospective comparative study	III	111	16 ± 1.4	44 ± 18	NR	Primary unilateral ACL‐rupture with no concomitant fractures.	ACLR (*n* = 40) ACLR + LEAT (*n* = 71)
Castoldi et al. [9]	France	Randomised Controlled Study	II	121	26 ± NR	233 ± NR	>94%	Primary unilateral ACL‐rupture and no concomitant ligament injury.	ACLR (*n* = 61) ACLR + LEAT (*n* = 60)
Gonnachon et al. [19]	France	Cohort study	III	79	22 ± 7.2	54 ± NR	NR	Primary unilateral ACL‐rupture & participating in pivoting sports.	ACLR (*n* = 39) ACLR + ALLR (*n* = 40)
Dejour et al. [12]	France	Retrospective comparative study	III	50	25 ± NR	NR	NR	Primary unilateral ACL‐rupture participating in sports	ACLR (*n* = 25) ACLR + LET (*n* = 25)
Perelli et al. [49]	Spain	Cohort study	III	66	141. ± 1.3	24 ± NR	NR	Pediatric patients with a primary ACL‐rupture and willingness to return to sport.	ACLR (*n* = 34) ACLR + LEAT (*n* = 32)

*Note*: All numerical data points are presented as mean and standard deviation unless marked with a * (these data points are medians with the range if provided by the respective article).

Abbreviations: 4HT, quadrupled hamstring tendon; ACL, anterior cruciate ligament; ACLR, anterior cruciate ligament reconstruction; ALLR, anterolateral ligament reconstruction; ALSA, anterolateral structure augmentation; LOE, level of evidence; LEAT, lateral extra‐articular tenodesis; MITBT, modified iliotibial band tenodesis; *N*, number of participants at the beginning of the study; NR, not reported.

### Surgical characteristics

The majority of ACLRs (96%) were performed using a (quadrupled) semitendinosus graft, sometimes combined with gracilis tendon. In one study [53], a bone‐patellar‐tendon‐bone (BPTB) graft was used. Regarding the concomitant lateral extra‐articular procedure, 10 articles reconstructed the ALL [11, 19, 20, 27, 34, 36, 41, 53, 54, 64]. In two studies augmentation of the anterolateral aspect of the knee was performed [10, 65] and in 12 studies LET was chosen [9, 12, 18, 28, 30, 39, 42, 49, 51, 59, 60]. Most of these LETs were performed according to the Lemaire (or slightly modified Lemaire) technique, utilising the ITB as graft [9, 18, 28, 30, 39, 49, 51]. One study performed LET according to the Lemaire technique with a gracilis tendon graft [12]. Three studies [18, 42, 59] performed an extra‐articular MacIntosh modified Coker‐Arnold procedure using the ITB as graft. Table 2 shows a summary of the surgical characteristics.

**Table 2:** Summary of the surgical details regarding the ACLR of each respective article.

Study (year)	ACLR technique	LEA‐procedure
Sonnery‐Cottet et al. [54]	ACLR using quadrupled ST and G as grafts	ALLR using double G as graft
Yang et al. [64]	ACLR using ST and G as grafts	ALLR using a tibialis autograft
Ye et al. [65]	DB‐ACLR using quadrupled G and ST as grafts	ALSA using the anterior PL tendon
Chen et al. [10]	DB‐ACLR using double ST and G tendons	ALSA using the anterior PL tendon
Joseph et al. [30]	ACLR using quadrupled ST and G as grafts	LET using modified Lemaire technique with ITB as graft
Sonnery‐Cottet et al. [53]	ACLR according to the BPTB method	ALLR using ST and G grafts
Ibrahim et al. [27]	ACLR using double ST as graft	ALLR using double G graft
Getgood and Moatshe [17]	ACLR using quadrupled ST and/or G as graft(s)	LET using modified Lemaire technique with ITB as graft
Hamido et al. [20]	ACLR using quadrupled ST as graft	ALLR using double G as graft
Mahmoud et al. [39]	ACLR using quadrupled ST and G as grafts	LET using the ITB as graft
Laboudi et al. [34]	SB ACLR using quadrupled ST as grafts	ALLR using G as graft
Viglietta et al. [60]	SB‐ACLR using double G and ST as grafts	Extraarticular MacIntosh modified Coker‐Arnold procedure using ITB as graft
Rowan et al. [51]	SB‐ACLR using doubled G and ST grafts	LET using the ITB as graft
Lee et al. [36]	SB‐ACLR using quadrupled ST and G as grafts.	ALLR using a tibialis allograft
Coquard et al. [11]	ACLR using quadrupled ST and G as graft	ALLR using a HT as graft
Porter and Shadbolt [50]	SB‐ACLR using quadrupled ST as graft	LIliotibial band tenodesis
Vadala et al. [59]	ACLR using quadrupled ST and G as graft	Extraarticular MacIntosh modified Coker‐Arnold procedure using ITB as graft
Mogos et al. [41]	ACLR using ST and G as grafts	ALLR using G as graft
Jacquet et al. [28]	ACLR using a quadrupled ST as graft	LET using double G as graft
Monaco et al. [42]	SB ACLR using quadrupled ST and G as grafts	Extraarticular MacIntosh modified Coker‐Arnold procedure using ITB as graft
Castoldi et al. [9]	ACLR according to the BTPB method	LET using G as graft
Gonnachon et al. [19]	ACLR using ST and G as grafts	ALLR using the ST as graft
Dejour et al. [12]	SB ACLR according to the BPTB method	LET using modified Lemaire technique with G as graft
Perelli et al. [49]	ACLR using quadrupled ST and/or G as graft(s)	LET using modified Lemaire technique

Abbreviations: ACLR, anterior cruciate ligament reconstruction; ALSA, anterolateral structure augmentation; BTPB, bone‐patellar‐tendon‐bone; DB, double bundle; G, gracilis tendon; ITB, iliotibial band; LET, lateral extra‐articular tenodesis; PL, peroneus longus tendon; SB, single bundle; ST, semitendinosus tendon.

### Clinical outcomes

#### Postoperative Tegner score

**Figure 4:**
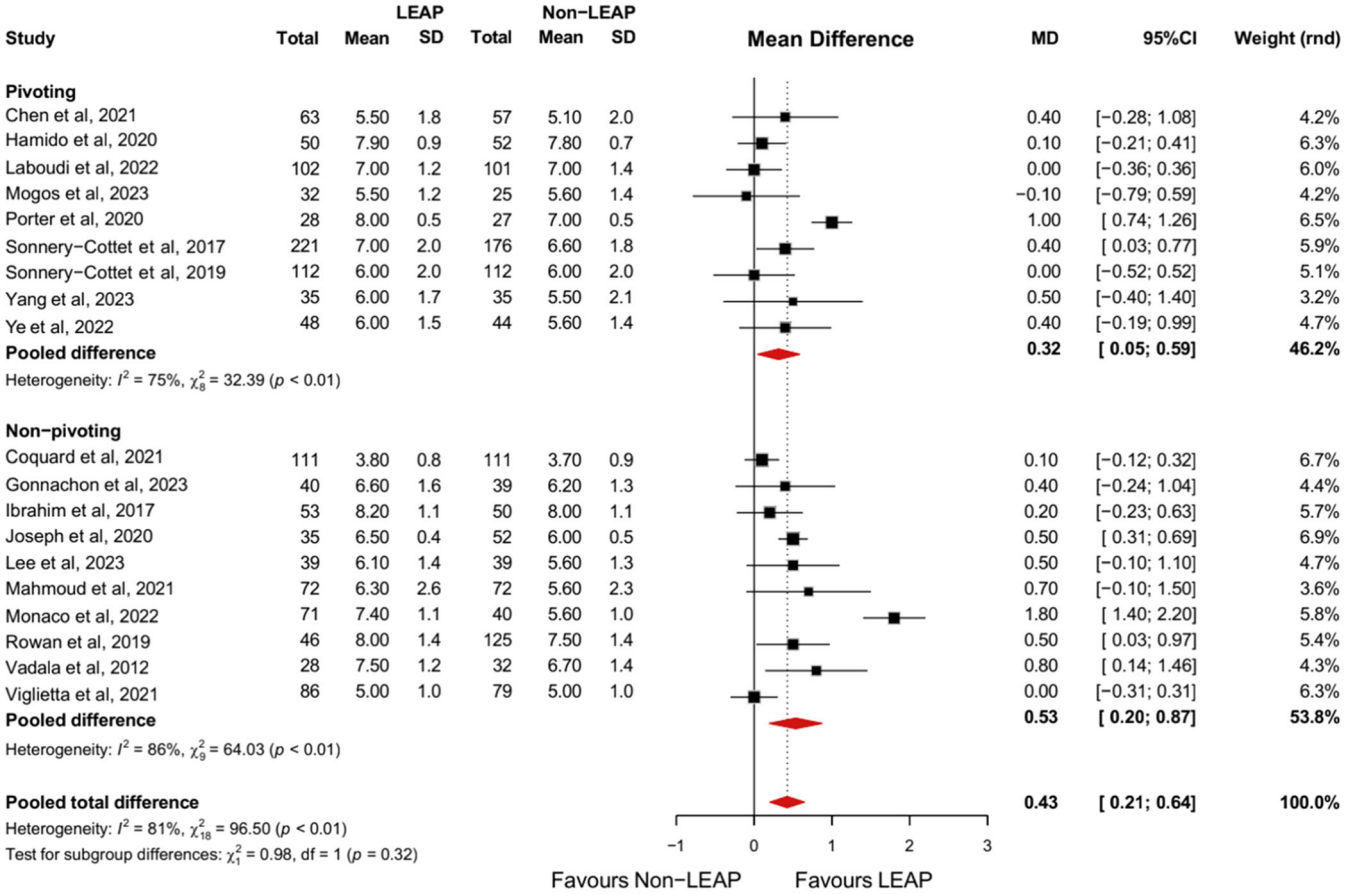
Forest plot of the comparison between the intervention and control group for the postoperative Tegner score with subgroup analyses comparing patients participating in pivoting‐ and non‐pivoting sports. 95%CI, 95% confidence interval; LEAP, lateral extra‐articular procedure; MD, mean difference; SD, standard deviation.

Nineteen studies reported a postoperative Tegner score [10, 11, 19, 20, 27, 30, 34, 36, 39, 41, 42, 50, 51, 53, 54, 59, 60, 64, 65]. The Tegner score is significantly higher in the population who received a concomitant LEAP alongside an ACLR than in the isolated ACLR group (MD, 0.43 [95% CI, 0.21–0.65]; *p* < 0.01). Subgroup analysis reveals that this applies to both the pivoting (MD, 0.32 [95% CI, 0.05–0.59]; *p* < 0.01) and non‐pivoting population (MD, 0.53 [95% CI, 0.20–0.87]; *p* < 0.01). The difference between these subgroups is not significant (Figure 4).

#### RTS

**Figure 5:**
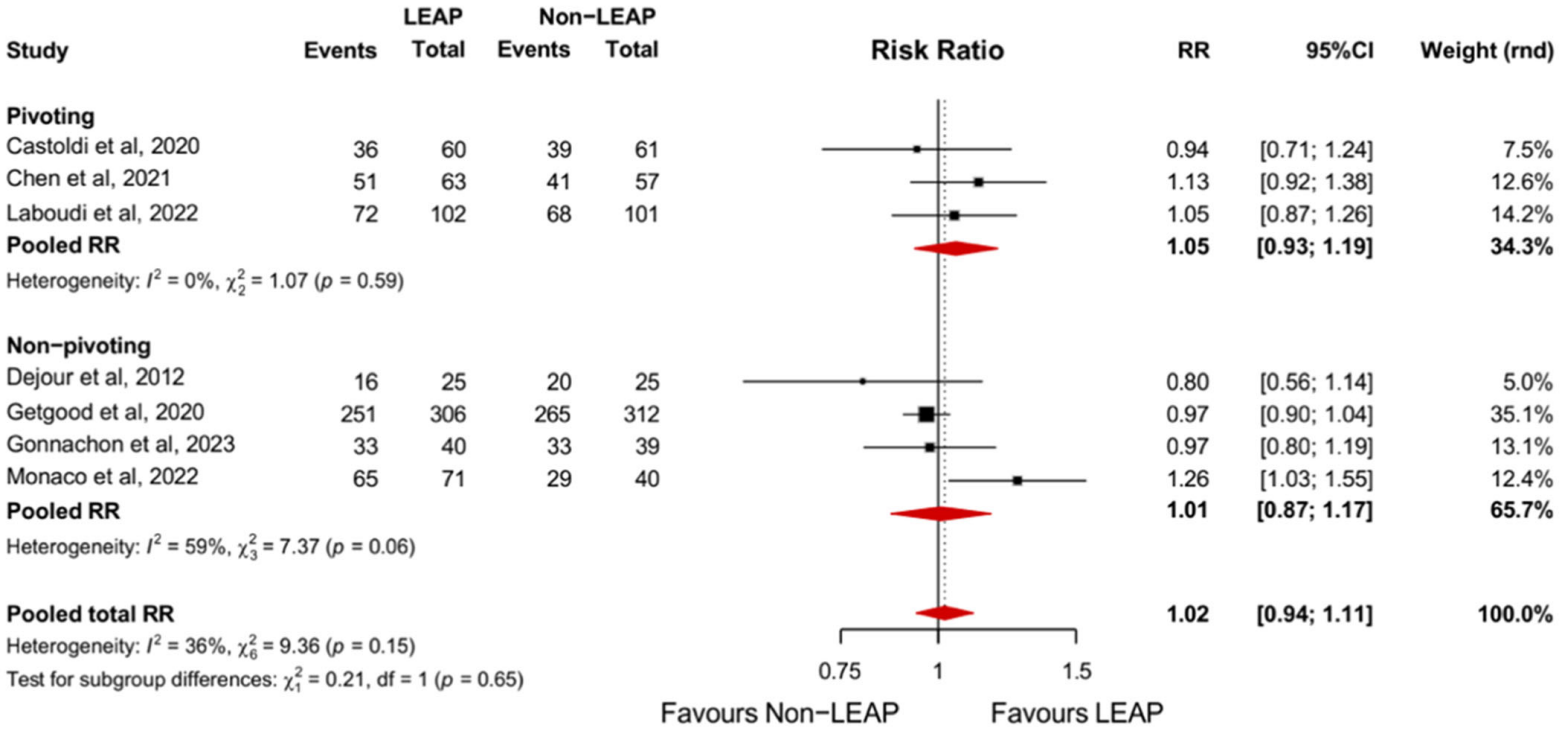
Forest plot comparing the intervention and control group based on the RTS with subgroup analyses comparing patients participating in pivoting‐ and non‐pivoting sports. 95%CI, 95% confidence interval; LEAP, lateral extra‐articular procedure; RR, risk ratio; RTS, return to sport.

Seven studies reported the RTS rate amongst their study population [9, 10, 12, 18, 19, 34, 42]. The likelihood to RTS is approximately equal between the ACLR + LEAP group‐ and isolated ACLR groups (RR, 1.02 [95% CI, 0.94–1.11]; *p* = 0.66). This was not different for subgroups: pivoting and non‐pivoting patients (Figure 5).

#### RTPS

**Figure 6:**
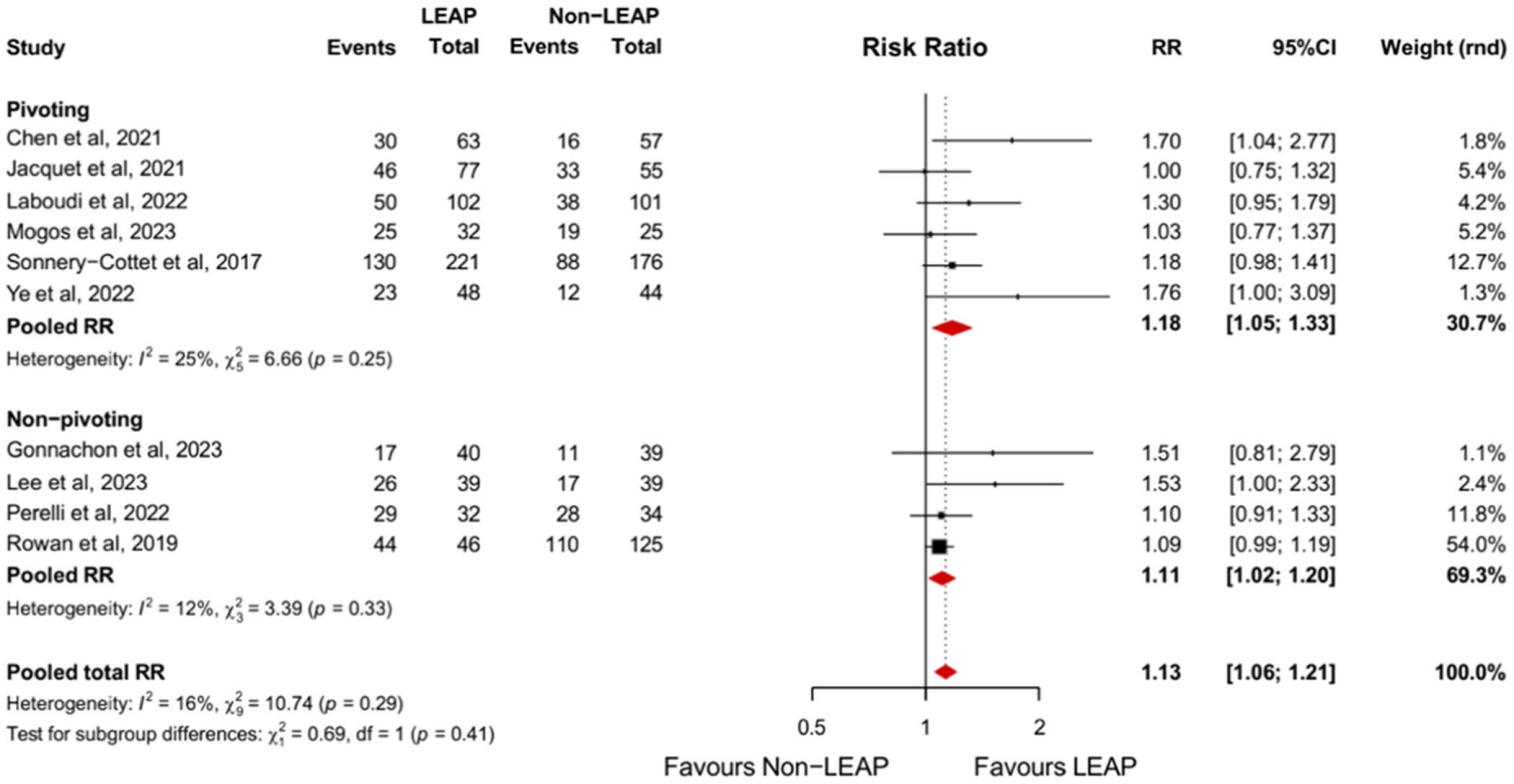
Forest plot comparing the intervention and control group based on the RTPS with subgroup analyses comparing patients participating in pivoting‐ and non‐pivoting sports. 95%CI, 95% confidence interval; LEAP, lateral extra‐articular procedure; RR, risk ratio; RTPS, return to pre‐injury‐level sport.

Ten studies reported the RTPS amongst their study population [10, 19, 28, 34, 36, 41, 49, 51, 54, 65]. The RTPS level is significantly higher in patients who received ACLR + LEAP than patients receiving ACLR alone (RR, 1.13 [95% CI, 1.06–1.21]; *p* < 0.01). This result is present in both the pivoting population (RR, 1.18 [95% CI, 1.05–1.33]; *p* < 0.01) and in the non‐pivoting population (RR, 1.11 [95% CI, 1.02–1.20]; *p* < 0.01). The difference in RTPS between the pivoting‐ and non‐pivoting subgroups is not significant (*p* = 0.41) (Figure 6).

#### Re‐rupture rate

**Figure 7:**
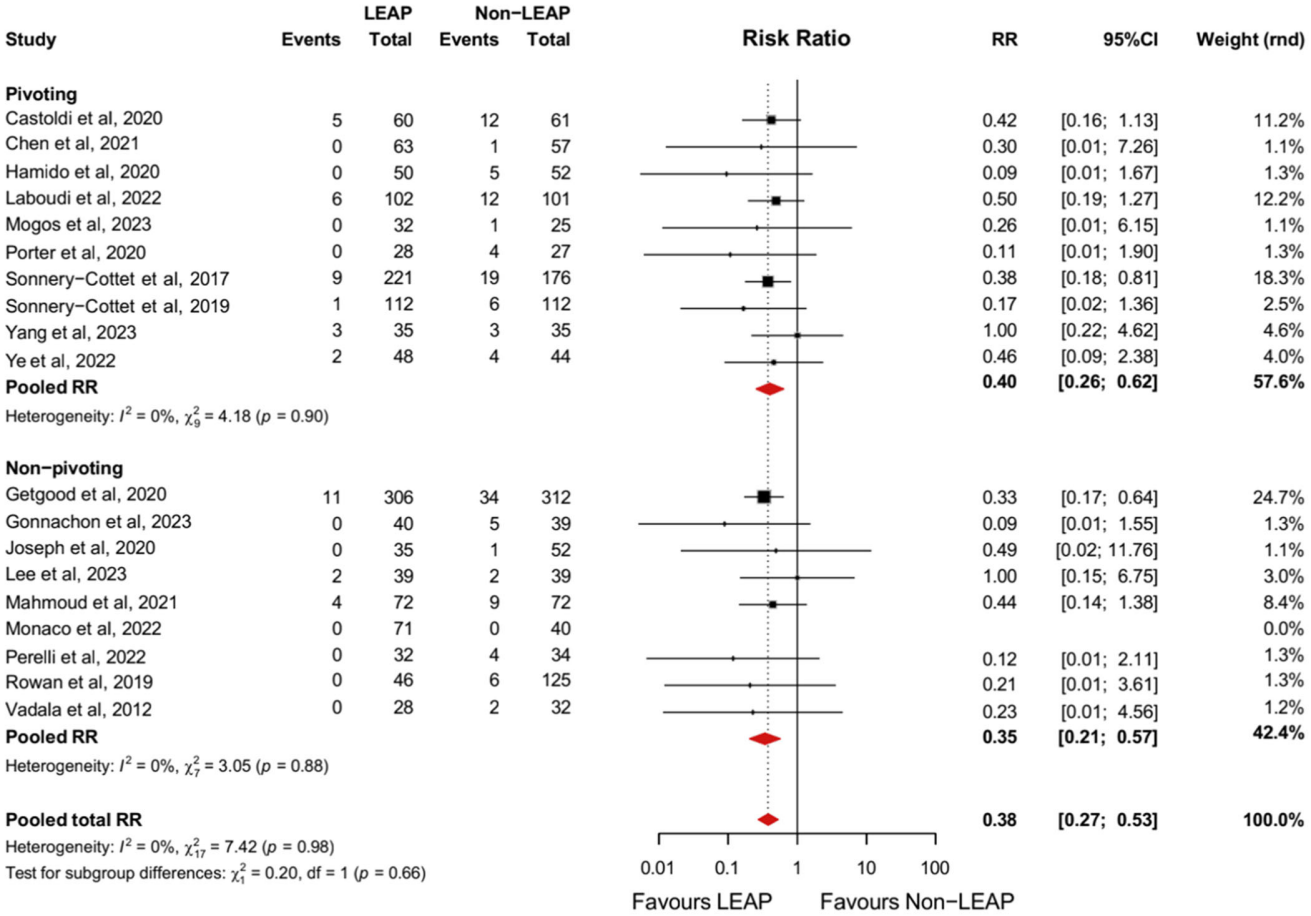
Forest plot comparing the intervention and control group based on the re‐rupture rate with subgroup analyses comparing patients participating in pivoting‐ and non‐pivoting sports. 95%CI, 95% confidence interval; LEAP, lateral extra‐articular procedure; RR, risk ratio.

Nineteen studies reported the re‐rupture rate amongst their study populations [9, 10, 18, 20, 30, 34, 36, 39, 41, 42, 49, 51, 53, 54, 59, 64, 65]. Patients who received a LEAP alongside an ACLR are significantly less likely to re‐rupture the same ACL than patients receiving an isolated ACLR (RR, 0.38 [95% CI, 0.27–0.53]; *p* < 0.01). This effect was also observed in the pivoting and non‐pivoting subgroups separately. A statistical difference between these subgroups was not observed (*p* = 0.66) (Figure 7).

#### Sensitivity analyses

Sensitivity analyses of the Tegner score, RTS, RTPS and re‐rupture rate showed no disparities amongst the results coming from RCTs and non‐RCTs. Further sensitivity analyses of RTS and RTPS proved that the data of these two outcomes is not impacted by missing data due to dropouts in all studies. See Tables 3, 4, 5, 6, 7, 8 for all sensitivity analyses.

**Table 3:** Sensitivity analyses comparing the results from RCT's with results from non‐RCT's on the postoperative Tegner score.

Tegner	No studies	Pooled MD (95%CI)	*p* value	*I* ^*2*^
Pivoting				
RCT	5	0.32 (−0.12; 0.75)	0.15	85%
Non‐RCT	4	0.26 (−0.01; 0.53)	0.06	1%
*Test subgroup difference*			0.81	
Non‐pivoting				
RCT	2	0.45 (−0.13; 1.02)	0.13	56%
Non‐RCT	8	0.55 (0.15–0.96)	0.01	89%
*Test subgroup difference*			0.77	

Abbreviations: MD, mean difference; RCT, randomised controlled trial.

**Table 4:** Sensitivity analyses comparing the results from RCT's with results from non‐RCT's on the return to pre‐injury level of sport.

RTS pre‐injury	No studies	Pooled RR (95%CI)	*p* value	*I* ^*2*^
Pivoting				
RCT	2	1.27 (0.78–2.06)	0.33	67%
Non‐RCT	4	1.18 (1.03–1.35)	0.02	18%
*Test subgroup difference*			0.78	
Non‐pivoting				
RCT	0	–	–	–
Non‐RCT	4	1.11 (1.02–1.20)	0.01	12%
*Test subgroup difference*			–	

Abbreviations: CI, confidence interval; RCT, randomised controlled trial; RR, risk ratio; RTS, return to sport.

**Table 5:** Sensitivity analyses comparing the results from RCT's with results from non‐RCT's on the return to sport.

RTS	No studies	Pooled RR (95%CI)	*p* value	*I* ^*2*^
Pivoting				
RCT	2	1.06 (0.89–1.25)	0.53	6%
Non‐RCT	1	1.05 (0.87–1.26)	0.62	NA
*Test subgroup difference*			0.96	
Non‐pivoting				
RCT	1	0.97 (0.90–1.04)	0.33	NA
Non‐RCT	3	1.02 (0.80–1.31)	0.85	67%
*Test subgroup difference*			0.66	

Abbreviations: CI, confidence interval; RCT, randomised controlled trial; RR, risk ratio; RTS, return to sport.

**Table 6:** Sensitivity analyses comparing the results from RCT's with results from non‐RCT's on the re‐rupture rate.

Rerupture	No studies	Pooled RR (95%CI)	*p* value	*I* ^*2*^
Pivoting				
RCT	6	0.29 (0.14–0.63)	<0.01	0%
Non‐RCT	4	0.47 (0.28–0.79)	<0.01	0%
*Test subgroup difference*			0.31	
Non‐pivoting				
RCT	2	0.32 (0.17–0.62)	<0.01	0%
Non‐RCT	7	0.39 (0.17–0.87)	0.02	0%
*Test subgroup difference*			0.74	

Abbreviations: CI, confidence interval; RCT, randomised controlled trial; RR, risk ratio.

**Table 7:** Sensitivity analyses comparing the results from the original sample size with results from population available at the last follow up (return to sport).

RTS	No studies	Pooled RR (95%CI)	*p* value	*I* ^2^
Original population				
Pivoting	3	1.05 (0.93–1.19)	0.40	0%
Non‐pivoting	4	1.01 (0.87–1.19)	0.92	59%
*Test subgroup difference*			0.65	
Population at FU				
Pivoting	3	1.05 (0.93–1.19)	0.40	0%
Non‐pivoting	4	0.98 (0.92–1.04)	0.74	0%
*Test subgroup difference*			0.27	

Abbreviations: CI, confidence interval; FU, follow‐up; RR, risk ratio; RTS, return to sport.

**Table 8:** Sensitivity analyses comparing the results from the original sample size with results from population available at the last follow up (return to pre‐injury level of sport).

RTS pre‐injury	No studies	Pooled RR (95%CI)	*p* value	*I* ^2^
Total population				
Pivoting	6	1.18 (1.05–1.33)	<0.01	25%
Non‐pivoting	4	1.11 (1.02–1.21)	0.01	12%
			0.42	
Population at FU				
Pivoting	6	1.16 (1.04–1.30)	<0.01	25%
Non‐pivoting	4	1.11 (1.02–1.20)	0.01	12%
*Test subgroup difference*			0.48	

Abbreviations: CI, confidence interval; RR, risk ratio; RTS, return to sport.

#### Funnel plot

**Figure 8:**
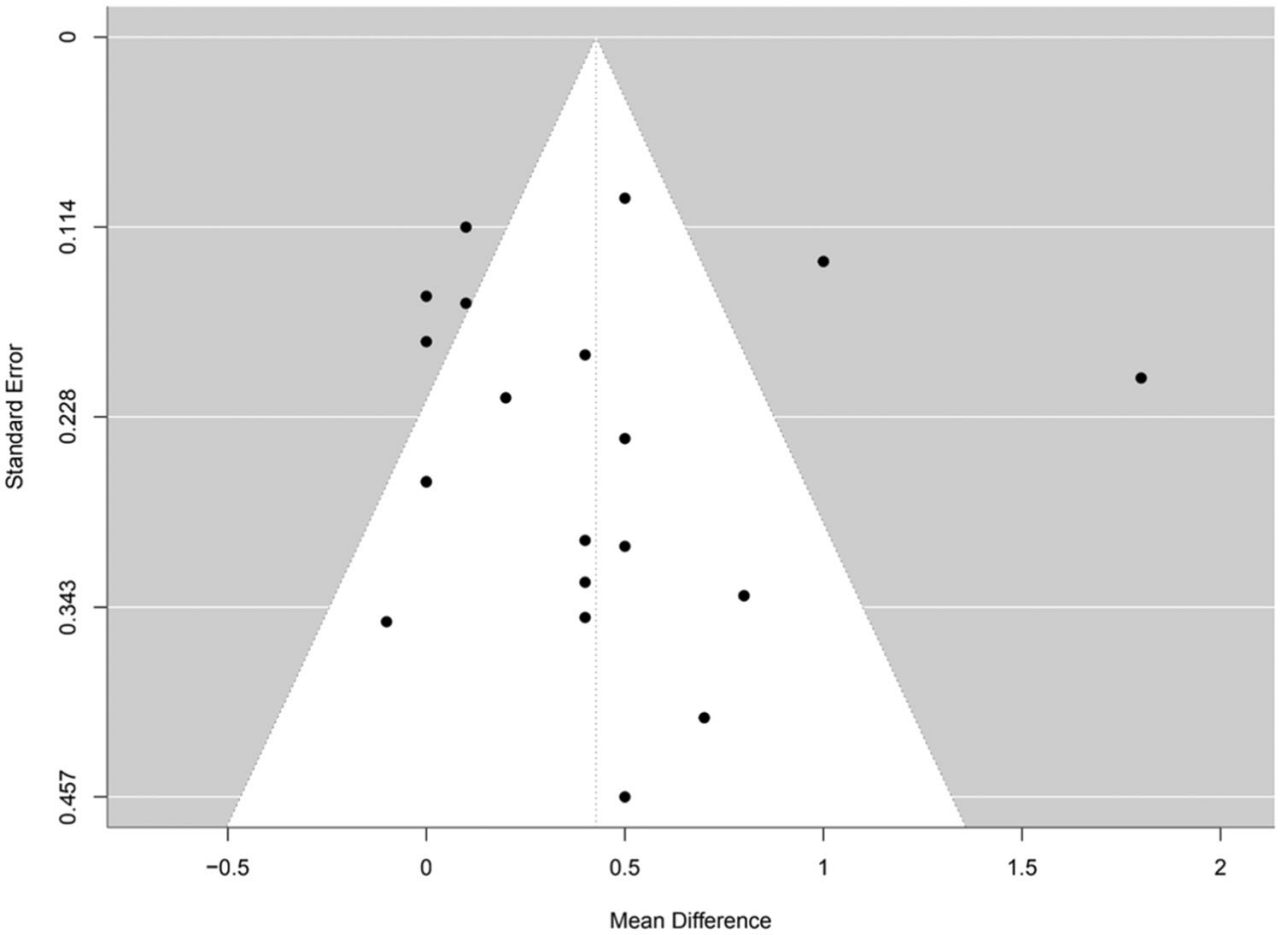
Funnel plot of the studies included in the Tegner score meta‐analysis.

The funnel plot reveals that the included studies are unevenly distributed indicating publication bias [57] (Figure 8).

## DISCUSSION

The most important finding of this study is that patients that underwent an ST‐tendon ALCR with a concomitant LEAP returned to a higher postoperative activity level than patients receiving an isolated ACLR. They also have a significantly higher chance of returning to their pre‐injury level of sport. This study did not find a difference between patients partaking in pivoting sports and patients involved in non‐pivoting sports. Furthermore, an additional LEAP leads to a lower risk of re‐rupturing the ACL‐graft. Given this growing body of evidence, LEAPs alongside ACL reconstructions may more and more become the (golden) standard of care.

From a biomechanical standpoint, the results of this study seem well explicable: as anterolateral reinforcement leads to more anterolateral rotatory stability, thus less residual pivot shift and thereby to a higher postoperative activity level [3]. Residual instability following an ACLR generally leads to lower activity levels [44].

The significantly higher postoperative activity level at the final follow‐up in the patient population receiving a LEAP is in accordance with other evidence. Na et al. [45] examined postoperative Tegner scores across 645 patients and concluded that LET and ALLR in addition to ACLR results in significantly higher activity levels at the final follow‐up, ranging from 6 months to 19 years. Regarding the RTPS; Beckers et al. [7] stated that the RTPS was 6% higher in the group who underwent lateral augmentation together with ACLR than the group who underwent ACLR alone. This review found that the RTPS was 22% higher in the ACLR + LEAP group than in the ACLR group. Unlike the RTPS, the RTS is reported more often within literature; and like this review Mao et al. [40] reported no statistical difference in RTS between patients receiving ACLR with‐ and without LET. The risk reduction with regards to re‐rupturing the ACL‐graft has been widely investigated. This significant risk reduction seems to exist across different LEAP techniques: ALL‐reconstruction [4], LET [18] and anterolateral augmentation [35].

Although partaking in pivoting sports is an important criterium for performing an additional LEAP to ACLR [53], subgroup analysis in this study was unable to demonstrate a greater benefit for the researched pivoting population. As described in the method of a case series paper by Sonnery‐Cottet et al. [55] certain criteria could dictate whether or not an ALLR is to be considered, such as: Grade III pivot shift, chronicity of the ACL rupture, high level of sports participation, age under 25, side‐to‐side laxity > 7 mm, associated Segond fracture, revision ACLR and participation in pivoting sports. Getgood and Moatshe [17] also concluded that participation in pivoting sports should contribute to the decision of operating extra‐articular as well as intra‐articular.

Additionally, according to Baker et al. [5] patient characteristics like increased posterior tibial slope (>9.5°) and knee hyperextension (>5°) predict a high risk of graft failure and thus extra‐articular reinforcement should be considered.

Since a lateral extra‐articular procedure significantly reduces the chance to re‐rupture the graft one might argue that *every* patient must receive a LEAP alongside an ACLR. Future research should also focus on patient‐related domains after an ACLR with a concomitant LEAP, such as: postoperative pain, range of motion, strength, coordination and knee‐related confidence. These aspects should be compared between patients receiving an isolated ACLR and patients receiving ACLR + LEAP. This may contribute to a more detailed and evidence‐based decision tree with regards to indications for LEAPs. Moreover, high‐quality evidence is required that compares the different LEAP‐techniques to further specify a future golden standard for treatment.

This review contains some limitations that were countered where possible. There was a high level of heterogeneity amongst the studies included in the Tegner score analysis. First, heterogeneity is caused by the differences amongst rehabilitation programs as these differ between countries and hospitals. Another major source of heterogeneity is the difference between the LEAP techniques and graft types amongst studies. However, in accordance with other literature, Van der Wal et al. [61] concluded that from a biomechanical aspect, it does not matter what kind of LEAP is combined with ACLR to ensure lateral extra‐articular reinforcement. This potential source of heterogeneity was therefore accepted to include relatively more articles compared to other systematic reviews [40, 52]. Therefore, the research question was not so much focused on *what* exact technique was used, but rather the effect of *any* (anterolateral) technique (on postoperative activity levels). Where the LEAP varied between the included studies, the ACLR‐technique did not as most studies performed a (quadrupled) ST graft. Thus, the conclusions of this review are most applicable for patients receiving this type of ACL graft.

RCTs and retrospective cohort studies were both included in this review. These different study designs with a different corresponding LOE may cause biased results. Sensitivity analyses showed however, that there is no difference in the effect when data from RCTs was compared to data from non‐RCTs. This corroborates the stability of the pooled estimates gathered from the meta‐analyses, which ensures that conclusions drawn are generalisable across the included studies.

One could argue that results regarding the primary outcome could have been presented as a change from baseline value. This would have involved the pre‐injury activity level of each group. However, the activity levels at last follow‐up between the two groups were analysed because a change of baseline data would likely have been inaccurate due to dropouts in all studies. Moreover, most of the included studies did not report a baseline activity level and thus a change of baseline value was not realistic. Overall pooling would have been based on different groups, due to patients being lost to follow‐up, which would have caused disparities amongst the results. Due to these arguments the Cochrane Institute advises authors to report post‐operative scores when drop‐outs are present [24].

Missing outcome data generates a level of bias within the results. All studies lost patients to follow‐up of which some due to re‐rupture; however, not one study explicitly performed an *intention‐to‐treat* analysis (ITT) which compensates for the missing data at last follow‐up. Therefore, a sensitivity analysis was performed that confirmed negligible influence of the data lost to follow up on the suggested effect. Also, there is a risk of selection bias influencing the results. Particular included studies [11, 27] assigned the intervention to patients who practice sport at a high level that likely have a strong willingness to return to their pre‐injury level of sport. This directly leads to biased results as the intervention was given to patients who were at baseline more likely to return to their pre‐injury level of sport. Simultaneously these patients may have followed extra rehabilitation programs and may have received additional professional guidance leading to further bias.

Then, there is publication bias. Figure 8 presents a funnel plot based on the included studies in the Tegner score meta‐analysis. The plot's asymmetrical character has several possible causations such as publication bias, poor methodological quality and heterogeneity [14]. Sterne et al. [57] stated that when a funnel plot is missing studies in the bottom left corner of the plot, publication bias is most likely the underlying cause of the asymmetry. Publication bias exists as statistically significant studies demonstrating a difference are more likely to be published. This directly leads to an overestimation of the true effect.

## CONCLUSION

The results of this meta‐analysis found that a LEAP in combination with a ST‐tendon ACLR leads to higher postoperative activity levels compared to an isolated ST‐tendon ACLR. Patients are also more likely to return to their pre‐injury level of sport. No significant difference was found to show that patients, involved in pivoting sports, benefit more than patients involved in non‐pivoting sports. Additionally, LEAPs lead to lower graft re‐rupture rates.

## AUTHOR CONTRIBUTIONS

**Guus Felix Kerkvliet**: Conceptualisation; methodology; systematic review and data collection; statistical analysis; writing—original draft preparation. **Gijs Bram Peter Cornelis van der Ree**: Systematic review and data collection. **Inger Nicoline Sierevelt**: Methodology; statistical analysis; review and editing; supervision. **Gino M. M. J. Kerkhoffs**: Review and editing; supervision. **Bart Muller**: Conceptualisation; methodology; review and editing; supervision All authors read and approved the final manuscript.

## CONFLICT OF INTEREST STATEMENT

The authors declare no conflicts of interest.

## ETHICS STATEMENT

There is no ethical approval necessary for this research paper.

## Supporting information

Supporting information.

## Data Availability

The authors confirm that the data supporting the findings of this study are available within the article and its supplementary materials.
