# Supplementary material for: Lateral extra‐articular procedures combined with ACL reconstructions lead to a higher return to pre‐injury level of sport: A systematic review and meta‐analysis
Source: J Exp Orthop. 2025 Mar 11;12(1):e70196. doi: 10.1002/jeo2.70196 (PMC11894468; doi:10.1002/jeo2.70196)
Supplement: Supplementary file 1 — Supporting information. [file JEO2-12-e70196-s001.docx]

# Additional files

**Complete search strategy (PubMed & Embase)**

**PubMed:**

# ("Anterior Cruciate Ligament"[Mesh] OR "Anterior Cruciate Ligament Reconstruction"[Mesh] OR "Anterior Cruciate Ligament Injuries"[Mesh] OR ACL tear*[tiab] OR ACL ruptur*[tiab] OR ACL injur*[tiab] OR ACL reconstruction*[tiab] OR anterior cruciate ligament reconstruction*[tiab] OR anterior cruciate ligament injur*[tiab] OR "Athletes"[Mesh] OR "Sports"[Mesh] OR athlete*[tiab] OR sport*[tiab])

# AND

# ("Tenodesis"[Mesh] OR lateral tenodes*[tiab] OR lateral extra articular tenodes*[tiab] OR anterolateral augmentation*[tiab] OR anterolateral ligament*[tiab] OR anterolateral complex*[tiab] OR anterolateral structur*[tiab] OR anterolateral ligament reconstruction*[tiab] OR modified Lemaire[tiab] OR Lemaire procedur*[tiab] OR Lemaire[tiab] OR extra-articular procedur*[tiab])

# AND

# ("Return to Sport"[Mesh] OR "Patient Reported Outcome Measures"[Mesh] OR patient reported outcome*[tiab] OR PROM[tiab] OR PROMs[tiab] OR clinical outcome*[tiab] OR Tegner[tiab] OR Tegner score*[tiab] OR return to sport*[tiab] OR return to play*[tiab] OR resumption to sport*[tiab] OR resumption to play*[tiab] OR pre-injur*[tiab] OR performance*[tiab] OR level[tiab] OR activity level*[tiab] OR pivoting sport*[tiab] OR pivot shift*[tiab] OR pivot shift test*[tiab])

# Embase (OVID):

| **#** | **Searches** | **Results** |
| --- | --- | --- |
| 1 | anterior cruciate ligament/ | 13935 |
| 2 | anterior cruciate ligament reconstruction/ | 15961 |
| 3 | exp anterior cruciate ligament injury/ | 13693 |
| 4 | ((anterior cruciate ligament* or ACL) adj3 (tear* or ruptur* or reconstruct* or injur*)).ti,ab,kf. | 26185 |
| 5 | exp athlete/ or exp sport/ or (athlete* or sport*).ti,ab,kf. | 337190 |
| 6 | 1 or 2 or 3 or 4 or 5 | 363546 |
| 7 | tenodesis/ | 2477 |
| 8 | (lateral tenodes* or lateral extra articular tenodes* or anterolateral augmentation* or anterolateral ligament* or anterolateral complex* or anterolateral structur* or anterolateral ligament reconstruction* or modified Lemaire or Lemaire procedur* or Lemaire or extra-articular procedur*).ti,ab,kf. | 1347 |
| 9 | 7 or 8 | 3480 |
| 10 | return to sport/ | 6103 |
| 11 | ((return* or resumption*) adj3 (sport* or play*)).ti,ab,kf. | 11846 |
| 12 | exp patient-reported outcome/ | 56451 |
| 13 | (patient reported outcome* or PROM or PROMs or clinical outcome* or Tegner or Tegner score* or pre-injur* or performance* or level or activity level* or pivoting sport* or pivot shift* or pivot shift test*).ti,ab,kf. | 5230898 |
| 14 | 10 or 11 or 12 or 13 | 5250120 |
| 15 | 6 and 9 and 14 | 834 |

# Risk of bias assessment:

#
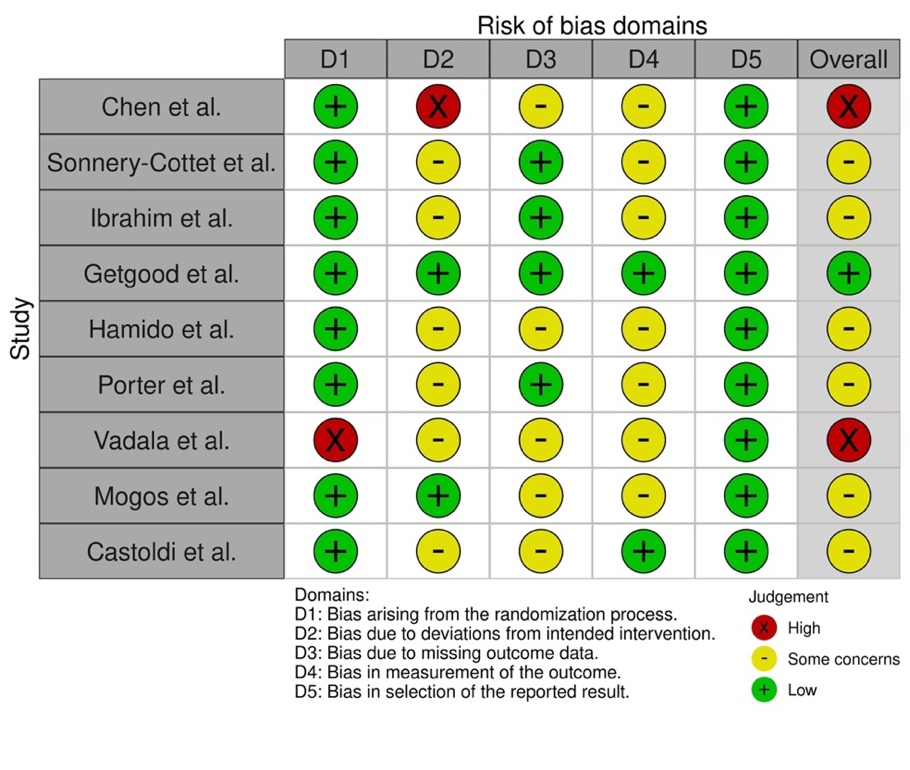


Figure 2: Critical appraisal of the included randomized controlled trials according to the RoB2 checklist


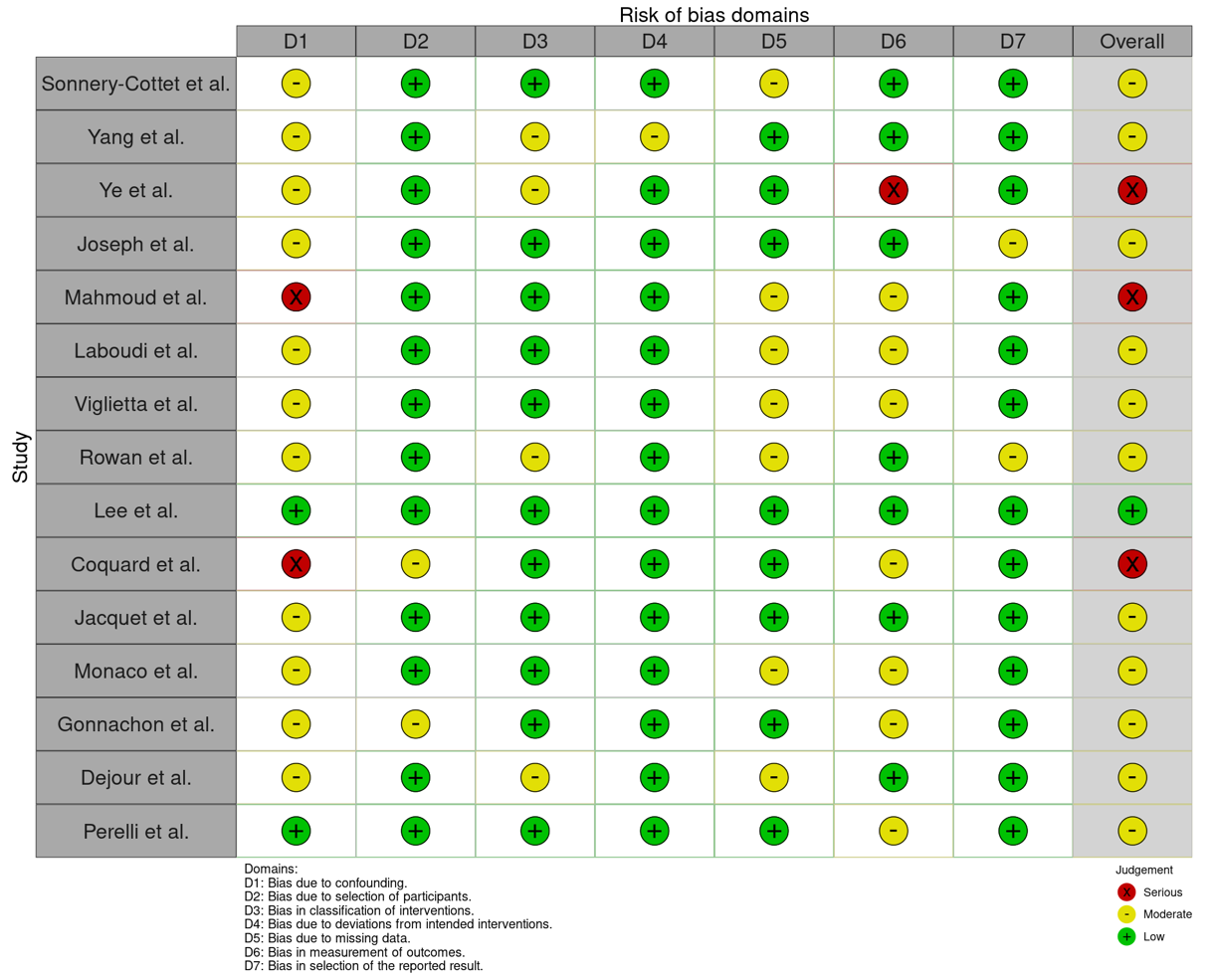


Figure 3: Critical appraisal of the included non-randomized controlled trials according to the ROBINS-1 checklist.

**Sensitivity analyses**

| **Tegner** | **No studies** | **Pooled MD (95%CI)** | **p-value** | ***I^2^*** |
| --- | --- | --- | --- | --- |
| **Pivoting** |  |  |  |  |
| RCT | 5 | 0.32 (-0.12; 0.75) | 0.15 | 85% |
| Non-RCT | 4 | 0.26 (-0.01; 0.53) | 0.06 | 1% |
| *Test subgroup difference* |  |  | 0.81 |  |
| **Non-pivoting** |  |  |  |  |
| RCT | 2 | 0.45 (-0.13; 1.02) | 0.13 | 56% |
| Non-RCT | 8 | 0.55 (0.15; 0.96) | 0.01 | 89% |
| *Test subgroup difference* |  |  | 0.77 |  |

Table 3: Sensitivity analyses comparing the results from RCT’s with results from non-RCT’s on the postoperative Tegner score.

| **RTS PRE INJURY** | **No studies** | **Pooled RR (95%CI)** | **p-value** | ***I^2^*** |
| --- | --- | --- | --- | --- |
| **Pivoting** |  |  |  |  |
| RCT | 2 | 1.27 (0.78; 2.06) | 0.33 | 67% |
| Non-RCT | 4 | 1.18 (1.03; 1.35) | 0.02 | 18% |
| *Test subgroup difference* |  |  | 0.78 |  |
| **Non-pivoting** |  |  |  |  |
| RCT | 0 | - | - | - |
| Non-RCT | 4 | 1.11 (1.02; 1.20) | 0.01 | 12% |
| *Test subgroup difference* |  |  | - |  |

Table 4: Sensitivity analyses comparing the results from RCT’s with results from non-RCT’s on the return to preinjury level of sport.

| **RTS** | **No studies** | **Pooled RR (95%CI)** | **p-value** | ***I^2^*** |
| --- | --- | --- | --- | --- |
| **Pivoting** |  |  |  |  |
| RCT | 2 | 1.06 (0.89; 1.25) | 0.53 | 6% |
| Non-RCT | 1 | 1.05 (0.87; 1.26) | 0.62 | NA |
| *Test subgroup difference* |  |  | 0.96 |  |
| **Non-pivoting** |  |  |  |  |
| RCT | 1 | 0.97 (0.90; 1.04) | 0.33 | NA |
| Non-RCT | 3 | 1.02 (0.80; 1.31) | 0.85 | 67% |
| *Test subgroup difference* |  |  | 0.66 |  |

Table 5: Sensitivity analyses comparing the results from RCT’s with results from non-RCT’s on the return to sport.

| **Rerupture** | **No studies** | Pooled RR (95%CI) | p-value | *I^2^* |
| --- | --- | --- | --- | --- |
| **Pivoting** |  |  |  |  |
| RCT | 6 | 0.29 (0.14; 0.63) | <0.01 | 0% |
| Non-RCT | 4 | 0.47 (0.28; 0.79) | <0.01 | 0% |
| *Test subgroup difference* |  |  | 0.31 |  |
| **Non-pivoting** |  |  |  |  |
| RCT | 2 | 0.32 (0.17; 0.62) | <0.01 | 0% |
| Non-RCT | 7 | 0.39 (0.17; 0.87) | 0.02 | 0% |
| *Test subgroup difference* |  |  | 0.74 |  |

Table 6: Sensitivity analyses comparing the results from RCT’s with results from non-RCT’s on the re-rupture rate.

| **RTS** | **No studies** | **Pooled RR (95%CI)** | **p-value** | ***I^2^*** |
| --- | --- | --- | --- | --- |
| **Original population** |  |  |  |  |
| Pivoting | 3 | 1.05 (0.93; 1.19) | 0.40 | 0% |
| Non-pivoting | 4 | 1.01 (0.87; 1.19) | 0.92 | 59% |
| *Test subgroup difference* |  |  | 0.65 |  |
| **Population at FU** |  |  |  |  |
| Pivoting | 3 | 1.05 (0.93; 1.19) | 0.40 | 0% |
| Non-pivoting | 4 | 0.98 (0.92; 1.04) | 0.74 | 0% |
| *Test subgroup difference* |  |  | 0.27 |  |

Table 7: Sensitivity analyses comparing the results from the original sample size with results from population available at the last follow up (return to sport).

| **RTS PRE INJURY** | **No studies** | **Pooled RR (95%CI)** | **p-value** | ***I^2^*** |
| --- | --- | --- | --- | --- |
| **Total population** |  |  |  |  |
| Pivoting | 6 | 1.18 (1.05; 1.33) | <0.01 | 25% |
| Non-pivoting | 4 | 1.11 (1.02; 1.21) | 0.01 | 12% |
|  |  |  | 0.42 |  |
| **Population at FU** |  |  |  |  |
| Pivoting | 6 | 1.16 (1.04; 1.30) | <0.01 | 25% |
| Non-pivoting | 4 | 1.11 (1.02; 1.20) | 0.01 | 12% |
| *Test subgroup difference* |  |  | 0.48 |  |

Table 8: Sensitivity analyses comparing the results from the original sample size with results from population available at the last follow up (return to preinjury level of sport).
